# A Novel Slope-Matrix-Graph Algorithm to Analyze Compositional Microbiome Data

**DOI:** 10.3390/microorganisms12091866

**Published:** 2024-09-09

**Authors:** Meng Zhang, Xiang Li, Adelumola Oladeinde, Michael Rothrock, Anthony Pokoo-Aikins, Gregory Zock

**Affiliations:** 1 Department of Mathematics, University of North Georgia, 82 College Cir, Dahlonega, GA 30597, USA; meng.zhang@ung.edu; 2 U.S. National Poultry Research Center, Egg & Poultry Production Safety Research Unit, Agricultural Research Service, U.S. Department of Agriculture, 950 College Station Road, Athens, GA 30605, USA; ade.oladeinde@usda.gov (A.O.); michael.rothrock@usda.gov (M.R.J.); gregzock@gmail.com (G.Z.); 3 U.S. National Poultry Research Center, Toxicology & Mycotoxin Research Unit, Agricultural Research Service, U.S. Department of Agriculture, 950 College Station Road, Athens, GA 30605, USA; anthony.pokoo-aikins@usda.gov

**Keywords:** microbiome, zero-inflated compositional data, rate of change, differential abundance analysis (DAA), graph theory, slope-based distances

## Abstract

Networks are widely used to represent relationships between objects, including microorganisms within ecosystems, based on high-throughput sequencing data. However, challenges arise with appropriate statistical algorithms, handling of rare taxa, excess zeros in compositional data, and interpretation. This work introduces a novel Slope-Matrix-Graph (SMG) algorithm to identify microbiome correlations primarily based on slope-based distance calculations. SMG effectively handles any proportion of zeros in compositional data and involves: (1) searching for correlated relationships (e.g., positive and negative directions of changes) based on a “target of interest” within a setting, and (2) quantifying graph changes via slope-based distances between objects. Evaluations on simulated datasets demonstrated SMG’s ability to accurately cluster microbes into distinct positive/negative correlation groups, outperforming methods like Bray–Curtis and SparCC in both sensitivity and specificity. Moreover, SMG demonstrated superior accuracy in detecting differential abundance (DA) compared to ZicoSeq and ANCOM-BC2, making it a robust tool for microbiome analysis. A key advantage is SMG’s natural capacity to analyze zero-inflated compositional data without transformations. Overall, this simple yet powerful algorithm holds promise for diverse microbiome analysis applications.

## 1. Introduction

Operational taxonomic units (OTUs) and sequence variants (SVs) are widely used to answer “who are they” and “how abundant are they” questions. Particularly, network analyses seem to be a tool to reveal microbiome correlations and clusters using OTUs and SVs. Networks, also known as graphs in mathematics, are widely used to infer relationships between objects [1]. For example, node-edge visualization networks are believed to efficiently demonstrate the interrelationships among different microorganisms within an environment. High-throughput sequencing has enabled the application of networks to hundreds of thousands of ecosystems; however, challenges such as sample handling, data preprocessing, and network interpretations are often overlooked [2]. Various statistical algorithms, such as Spearman rank correlations and the Bray–Curtis dissimilarity, are widely used to interpret network edges linked to the nodes [2,3]. Nonetheless, many biologists apply software tools/packages directly without considering the limitations of these algorithms. For instance, Zhang et al. (2016) [4] discussed the serious drawbacks of the Spearman rank correlation network, which has been widely cited in microbiome research [5].

Concerns about the inappropriate use of network analysis have recently arisen. For instance, as described in depth in Faust (2021) [2], rare taxa require attention when constructing networks. Particularly, analyzing large proportions of rare taxa in a study becomes even more challenging when simultaneously examining over 11,000 bacterial species from 500 amplicon sequencing samples [6]. In addition, the interpretations of compositional microbiome sequencing data need more attention when dealing with relative abundance [7,8]. For example, the term “observed abundance” can be confusing, making data analyses challenging for researchers without a background in statistics [9]. Essentially, high-throughput sequencing-related compositional data analysis starts with normalization, which Weiss et al. (2017) demonstrated rarefying outperformed the other scaling methods (e.g., log upper quartile, cumulative sum scaling, and variance stabilization) [7]. The purpose of normalization is to transform data in order to enable accurate comparison of statistics from different measurements. Post-normalized data then proceed to differential abundance analysis (DAA) that focuses on between-group comparisons (e.g., treatment vs. control). Arguably, just like choosing normalization methods, researchers also need to pay attention to selecting appropriate algorithms [7]. For instance, non-parametric methods are often preferred because OTUs and SVs do not follow normal distributions [7]. However, non-parametric methods generally cannot handle the compositional characteristics of data, making them inapplicable. Weiss et al. (2017) systematically compared some widely used DAA algorithms (e.g., the Mann–Whitney U test, DESeq2, edgeR, analysis of composition of microbiomes (ANCOM), and metagenomeSeq), and their results suggest ANCOM has a lower false discovery rate (FDR), but it is only sensitive given > 20 samples/group [7].

Graph theory (GT) studies graphs, which are structures used to learn relations between objects [10]. Finding dense subgraphs, which are clusters in a network, and using vertex cuts to label the control points are common methods used in analyzing biological relationships [11]. For instance, after sequencing bacterial pathogen-related samples from an environmental sample, researchers are eager to know which samples are pathogenic, how abundant they are within the setting, which microbes may relate to those pathogens, and whether there is any microbial community cluster within this specific niche. During preliminary data analysis, researchers may notice different magnitudes of abundances across multiple samples; however, the changing patterns are still similar, either increasing or decreasing simultaneously among samples. The pattern of increasing or decreasing can be explained by the rate of change in mathematics. Given that the samples in each taxon are inherently discrete and have no order, the rates of changes between two samples are equivalent to the slope of a straight line. Unlike only examining the values of individual taxa, analyzing the rates of changes can effectively reflect the structural role within a dataset.

Here, we introduce a simple and easy-to-use slope-based algorithm, Slope-Matrix-Graph (SMG), to quickly identify interrelationships within microbiome samples and to perform differential abundance analysis. SMG can deal with any proportion of zeros in compositional microbiome data, which is a concern for most current algorithms [9]. Our approach involves: (1) searching for correlated interrelationships based on any “target of interest”, and (2) calculating the magnitude of change between graphs (e.g., comparing microbiomes after different treatments) through the analysis of the slope-based distance. The overarching goal is to provide biologists with simple, fast, and confident interpretations of complex high-throughput sequencing data, such as amplicon and metagenomic sequencing data.

## 2. Materials and Methods

### 2.1. Slope-Matrix-Graph (SMG) Algorithm Introduction

#### 2.1.1. Slope-Based Distance Function Definition

The rate of change between any two values, *u* and *v*, is u−v. It is equivalent to the slope of a linear function with points (w, u) and (w+1, v), where *w* can be any number. Now, we can extend it to the case of two objects with *n* samples. Let $A=\{a_{1},\;\ldots ,\;a_{n}\}$ and $B=\{b_{1},\;\ldots ,\;b_{n}\}$ be the abundance sets of two objects across n samples, where $a_{k}$ and $b_{k}$ represent the respective abundances of the two objects in the *k*-th sample $\left(k\in \left\{1,\;\ldots ,\;n\right\}\right)$. To keep the range of value in each object as [0, 1], we use min–max feature scaling for abundance of objects. So, we have

$$\alpha_{k}=\left\{\begin{array}{c} \frac{a_{k}-\min A}{\max A-\min A},\;if\;\min A\neq \max A\\ 0,\;if\min A=\max A \end{array}\right.,\;\beta_{k}=\left\{\begin{array}{c} \frac{b_{k}-\min B}{\max B-\min B},\;if\;\min B\neq \max B\\ 0,\;if\min B=\max B \end{array}\right.,$$

where $\left(\min X,\max X\right)=\left\{\left(y,z\right)\in X\times X\right|\;y\leq x\leq z\;forany\;x\in X\}$ and X∈{A, B}. Then, for any $i,j\in \left\{1,\;\ldots ,\;n\right\}$, the difference of slope among *i*-th and *j*-th samples between objects *A* and *B* is $\left|\left(\alpha_{j}-\alpha_{i}\right)-\left(\beta_{j}-\beta_{i}\right)\right|$. So, our distance function which can present the difference of slope between two objects *A* and *B* can be defined as

$$d\left(A,\;B\right)=\sum_{\begin{array}{c} 1\leq i\leq n\\ 1\leq j\leq n \end{array}}\left|\left(\alpha_{j}-\alpha_{i}\right)-\left(\beta_{j}-\beta_{i}\right)\right|.$$

**Figure 1:**
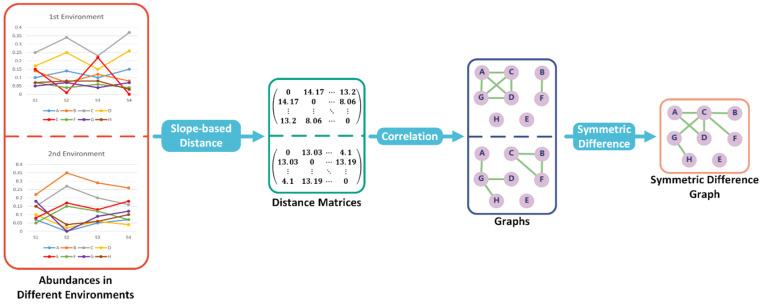
Overview of SMG’s differential abundance analysis (DAA) algorithm. The distance matrix can be calculated using the slope-based distance function, and to contrast the disparities among objects in two environments, we determine the symmetric difference between two graphs based on the calculated correlations. In the end, the edges in symmetric difference graphs are the positive correlations that have changed from one environment to another.

Subsequently, a distance matrix can be obtained such that each entry represents the distance between two associated objects (see Figure 1).

#### 2.1.2. Positive and Negative Correlation Definitions

If the distance between two objects is small enough, their difference in slope is small. In other words, their abundance values exhibit similar behavior, and we call that “exhibiting a positive correlation”. To define the indicative value of distance for a positive correlation, our approach involves identifying two distinct, indicative objects that are separated from each other, ensuring no other object simultaneously exhibits the same level of correlation with both. Assume we have n samples in each object. So, each object corresponds to a point in n-dimensional space. We can set our first indicative object as O={0, 0, …, 0}, which corresponds to the origin point in the *n*-dimensional space. Since the range of value in each object is [0, 1] after we use min–max feature scaling, as long as the original abundance of the object does not consist only of 0 s, then the scaled object contains at least one 1. The second indicative object that satisfies the above conditions, and whose associated point is the closest to the origin in n-dimensional space, should be P={1, 0, …, 0}. Notably, the associated point of P is the unit point on the first coordinate axis in the same space. Our objective is to ensure that all other objects have a positive correlation with one of *O* and *P* at most. Since d(O, P)=2(n−1), it implies the half of d(O, P), which is *n* – 1, can satisfy our objective, as the threshold for defining the bounds of positive correlation. In other words, in the context of *n* samples, if the distance between two organisms is less than *n* − 1, it indicates a positive correlation between them; otherwise, the positive correlation is considered absent. For negative correlation, we are looking for an object which has a positive correlation with the opposite of the target object. Suppose $A=\{a_{1},\;\ldots ,\;a_{n}\}$ is the target object. We set the opposite of *A* as $A^{-}=\{-a_{1},\;\ldots ,-a_{n}\}$. If there exists an object *B* with $d\left(A^{-},\;B\right)<n-1$, which means *B* has a positive correlation with $A^{-}$, then we say *B* has a negative correlation with *A*.

#### 2.1.3. Graphs and Clusters

Suppose there is a non-empty set V(G). Let E(G) be a subset of the set of all unordered pairs of elements in V(G). Then we call the ordered pair (V(G),E(G)) as a graph *G*, where V(G) is the vertex set and E(G) is the edge set. Let *u* be a vertex in graph G. There is an edge subset *D* such that any edge in *D* has *u* as one end. Then the degree of *u*, which is *d*(*u*), equals the size of *D*. Moreover, a graph H=(V′,E′) is called a subgraph of *G* if V′ is a subset of V(G) and any edge in E′ which is a subset of E(G) has both ends in V′. A connected component, *C* of graph *G*, is a connected subgraph and satisfies the requirement that there is a path which is an interconnected sequence of edges within *C* that connects two vertices if and only if these two vertices are both in *C* (see Figure 1). In other words, a connected component is a cluster of objects in a biological network.

#### 2.1.4. Symmetric Difference between Two Graphs

Suppose $G_{1},\;G_{2}$ are two graphs with the same vertex set *V*. We denote the symmetric difference between $G_{1}$ and $G_{2}$ as $G_{1}∆G_{2}$ which is a graph with vertex set *V* and edge set $E\left(G_{1}∆G_{2}\right)=\left[E\left(G_{1}\right)\cup E\left(G_{2}\right)\right]-\left[E\left(G_{1}\right)\cap E\left(G_{2}\right)\right]$, where $E\left(G_{1}\right)$ or $E\left(G_{2}\right)$ is the edge set of $G_{1}$ or $G_{2}$, respectively. The magnitude of change between $G_{1}$ and $G_{2}$ is given by the number of edges in $G_{1}∆G_{2}$, denoted as $\left|E\left(G_{1}∆G_{2}\right)\right|$.

#### 2.1.5. Comparison Filter with Proportional Zeroing

Suppose a data set contains n taxa, $T_{1},\;T_{2},\;\ldots ,\;T_{n}$. Let $t_{i}$ be the sum of abundance across all samples for taxa $T_{i}$ (1≤i≤n) and $t=\sum_{i=1}^{n}t_{i}$. We sort $t_{1},\;t_{2},\ldots ,\;t_{n}$ from largest to smallest to obtain a sequence $t_{1}^{*},\;t_{2}^{*},\;\ldots ,\;t_{n}^{*}$, where $t_{i}^{*}$ is associated with taxa $T_{j}$ (1≤j≤n). Let *p* be the proportion of a data set we wish to retain, where 0%≤p≤100%. We can find m such that m≤n with

(1) $$\sum_{i=1}^{m}t_{i}^{*}\geq p*t\;\mathrm{and}\;\sum_{i=1}^{m-1}t_{i}^{*}<p*t$$

Thus, the taxa associated with $t_{1}^{*},\;\ldots ,\;t_{m}^{*}$ are those we retain based on filter *p*. In other words, we keep the largest taxa whose total proportion in the overall data is at least *p*. In this study, we set the starting point for *p* at 95% and performed an analysis for every 0.5% increment until reaching 100%. For the data, which is out of the comparison filter, we use the method called proportional zeroing to adjust the value of all samples into zeros.

### 2.2. Real-World Application and Simulation Data Description

Zou et al. (2022) conducted a systematic investigation into the composition and functionality of chicken gut microbiota in response to antibiotic growth promoters (AGPs) [12]. Briefly, we applied SMG symmetric difference function (Section 2.1.4) on their OTU data (DESeq2 normalized, refer to Supplementary File S1), and both Day 24 and Day 40 of the post-AGP sequencing data were analyzed. One of the primary conclusions was that more taxa in the chicken ceca were affected by dietary changes than by the application of AGPs [12]. Our analysis began with the Day 24 data. We applied the comparison filter (Section 2.1.5) starting at 95% and increasing by 0.5% up to 100%. For each comparison filter setting, we applied the slope-based distance function on all pairs of OTUs (Section 2.1.1). Distance matrices were generated for different feeding conditions, and correlation graphs were constructed by applying *n* − 1 as the threshold for positive correlation (Section 2.1.2), where n was the number of samples in the feeding condition. Subsequently, we calculated the number of edges in the symmetric difference of the graphs (Section 2.1.4) for four pairs: corn only vs. wheat only, corn with AGP vs. wheat with AGP, corn only vs. corn with AGP, and wheat only vs. wheat with AGP.

Furthermore, we reanalyzed data from Sylvain et al. (2019) [13], who examined the differences in bacterial community structures between the gut and skin mucus of two Amazonian fish species. One of their key findings was that the bacterial communities in the gut exhibited more connections compared to those on the skin mucus, indicating a denser bacterial network in the gut. Using their data (refer to Supplementary File S4), we categorized five groups within both the gut and skin mucus datasets: flag cichlids from blackwater, mixed water, and whitewater, and black piranhas from blackwater and whitewater. After removing SVs with zero values across all samples in each category, we calculated the distance between each pair of non-zero SVs using the slope-based distance function (Section 2.1.1) and constructed the corresponding distance matrices. Positive correlations were determined by setting a threshold of *n* − 1, where n is the number of samples in each category, to generate the relevant graphs (Section 2.1.2). Finally, we assessed the complexity of each network by calculating the average degree of each graph, defined as the sum of all vertex degrees divided by the number of vertices.

Tito et al. (2024) [14] conducted a study on the fecal microbiota of 589 patients across three colorectal cancer (CRC) stages: CRC, CTL (patients without evidence of colonic lesions), and ADE (patients with polyps). Using quantitative microbiome profiling (QMP) and relative microbiome profiling (RMP) based on 16S ribosomal RNA amplicon sequencing, they identified several stable CRC biomarkers. We reanalyzed the absolute abundance data from this study (see Supplementary File S5) to compare SMG with QMP. Briefly, we first applied a comparison filter with proportional zeroing (Section 2.1.5) across the three categories. Next, we calculated the pairwise distances between taxa within each category using the slope-based distance function (Section 2.1.1) under different comparison filters, constructing the corresponding distance matrices. Finally, we set the threshold for positive correlation as *n* − 1 (Section 2.1.2) to compare the groups, specifically CTL vs. CRC and ADE vs. CRC, where n represents the sample size within each category. 

**Figure 2:**
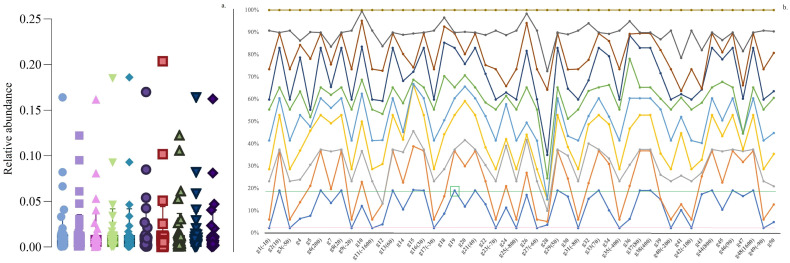
Simulation data type 1 example (50 rows × 10 columns) of a relative abundance matrix. (**a**) represents the distribution of relative abundance values from 10 columns (colored with different shapes); the horizontal axis of (**b**) represents rows; and the colored dot represents the distributions of the relative abundances from 10 columns. In our design, G19 is the “target of interest”, and both “positive” and “negative” stimulations are obtained by (1) “positive” equals the coefficient × G19 (parentheses include the percentage coefficient value, e.g., 800 represents a coefficient of 8); and (2) “negative” equals the coefficient × (1 − G19), with a “−” sign used as an indication of negative correlation manipulations. G19 is green-framed, with the corresponding horizontal green line representing the relative abundance threshold; the pink line shows the negative correlation threshold across all 50 rows.

Additionally, we analyzed two simulated data types, evaluating SMG correlation and graph change functionalities, respectively (Section 2.1.3 and Section 2.1.4). The first type of simulation data consisted of 10 replicates of balanced compositional data [15]. These datasets were structured as five 50 × 10 and five 100 × 20 matrices (e.g., representing 100 annotated microbes from 20 samples in a setting). Particularly, one row was designated as the “target of interest” (G19 in Figure 2 and Figure S1). Briefly, “positive correlation” was firstly defined as the product of a coefficient and G19, while “negative correlation” was calculated as the coefficient × (1 − G19) (see Figure 1 caption for more details). Notably, we utilized relative abundance (summing to 100%) to generate random numbers for simulation data type 1 (refer to Supplementary File S2). 

For the second simulation data type, we designed five DAA (100 × 5 matrices, between-group comparison), ranging from 3% to 20% (see Supplementary File S3 for more details). In brief, we modified the “GUniFrac” package in R (version 4.3.1) to simulate our type 2 OTU data (*SimulateMSeq* function) [15]. This data type focused on differential OTUs from a mixture of both the top and bottom quartiles of the abundance distributions, with the direction of change set as “balanced” [15]. Essentially, we manually incorporated between-group differences by controlling the DAA proportions at 3%, 5%, 7%, 10%, and 20%, with 100 taxonomic classifications forming two groups, each consisting of 5 samples.

### 2.3. Method Evaluations

In addition to our SMG algorithm, we utilized the Bray–Curtis dissimilarity and sparse correlations for compositional data (SparCC) methods [16,17] on the simulation data type 1. These algorithms are widely recognized for inferring microbial ecological interactions from microbial abundances. Specifically, PAST (PAleontological STatistics) (version 4.03) was utilized to calculate Bray–Curtis dissimilarity (Fruchterman–Reingold algorithm with an edge cutoff at 80%).

For simulation data type 2, designed to study DAA, we compared SMG to ANCOM-BC2 [18] and ZicoSeq [15], which have been shown to outperform other alternatives in the field currently. In brief, we used the R packages “ANCOMBC”, “dplyr”, “tidyr”, and “phyloseq” to perform ANCOM. For ZicoSeq, the “GUniFrac” R package was applied, and OTU count was used for the *feature.dat.type* parameter.

## 3. Results

### 3.1. Bray–Curtis Dissimilarity, SparCC, and SMG Evaluations on Simulation Data Type 1

One of the purposes of using balanced compositional simulation data type 1 is to determine whether the designated correlation “rows” can be identified when mixed with other signals at various “columns”. For instance, let the 50 × 10 matrix simulate 50 bacterial orders from 10 experimental runs/samples (*n* = 10). Ideally, we aim to divide the bacteria into several sets such that each set contains at least one bacterium positively correlated with any other bacterium in the same set, while any bacterium outside the set is not positively correlated with any bacterium inside the set. In simulation data type 1, the ideal scenario is to group all the bacteria preset to be positively correlated with G19 into one set and all negatively correlated bacteria into another set. As shown in Figure 2a (an example of relative abundances from a 50 × 10 matrix), most of the dense abundances were distributed below 0.05, with some high values near 0.20. Notably, Figure 2b illustrates that the relative abundance data with the same direction changes remained horizontally unchanged.

As detailed in Table 1, the Bray–Curtis dissimilarity method illustrated distinct cluster formation solely for coefficients ranging from 0.1 to 1, evident under the 80% edge cutoff. Notably, this method consistently produced cohesive results across all 10 simulations. In contrast, the default SparCC program (https://github.com/Labevo/MicNetToolbox, accessed on 7 August 2024) not only clustered coefficients within the 0.1 to 1 range, but also encompassed all specified positive correlations (2, 4, 8, and 16). Despite SparCC’s proficiency in capturing negative correlations, it’s important to note the presence of “random” data points in both positive and negative clusters. Conversely, our slope-based algorithm effectively delineated clear positive and negative clusters by incorporating all designated coefficients. Furthermore, robustness testing confirmed the clustering efficacy. The SparCC algorithm did not generate any clusters in two 50 × 10 simulation matrices.

### 3.2. DAA Using ANCOM-BC2, ZicoSeq, and SMG

Our simulation type 2 focuses on between-group comparisons and identifying designated OTU changes. As shown in Table 2, ZicoSeq is proficient in detecting differential abundance (DA) greater than 7%, while ANCOM-BC2 performs extremely well at relatively lower DA percentages, achieving over 99% accuracy for DA less than 10%. Utilizing SMG, we can achieve similar results (filtered out < 5% edge changes in total, see Section 2.1.4). Unlike ZicoSeq and ANCOM-BC2, which generate adjusted FDR *p*-values, SMG generates graph edge change counts. Interestingly, SMG’s accuracy is greatly impacted by the filter threshold (Section 2.1.5). As seen in Table S1, when the filter was set to 0.985, the accuracy dropped from 100% to 0% for 3% DA (with a similar trend observed for 3% to 10% DA). On the other hand, increasing the filter threshold to 0.955 is necessary to reach 100% accuracy for 20% DA.

### 3.3. Implementation of SMG on Previously Published Data

For the chicken gut microbiota in response to AGPs, as seen in Table 3A, with a 95% comparison filter, the symmetric difference between graphs for corn-only and wheat-only feeding conditions shows 10,800 edges. This indicates that 10,800 correlations among all OTUs either appear or disappear when switching from corn-only to wheat-only feeding. Table 3A consistently shows that, irrespective of the comparison filter, the sum of the edges in symmetric difference graphs due to dietary changes is always greater than those due to AGP changes, ranging from 5890 to 2106. Given that there are 256 OTUs in the dataset, our analysis indicates that, on average, each OTU experienced an increased change in its interrelation with other OTUs from a high of 23.01 to a low of 8.23 OTUs when dietary conditions were altered compared to the addition of AGP in the Day 24 dataset. A similar result is obtained for the Day 40 dataset. Table 3B indicates that, with comparison filters ranging from 95% to 100% at 0.5% increments, the sum of the number of edges in symmetric difference graphs due to dietary changes consistently exceeds those due to AGP changes, ranging from a high of 6860 to a low of 212. This suggests that, on average, each OTU experienced an increased change in its interrelation with other OTUs from a high of 26.8 to a low of 0.83 OTUs when dietary conditions were altered compared to the addition of AGP in the Day 40 dataset. Therefore, using SMG, we also demonstrated that altering the diet of chickens has a greater impact on the bacterial flora in the chicken cecum than changes in AGP levels.

When reanalyzing the data from Sylvain et al. (2019) [13], we found that the gut bacterial communities of flag cichlids in black water contained 698 non-zero SVs, with an average degree of 31.143. In brief, this average degree of 31.143 suggested each non-zero SV in this network was positively correlated with 31.143 other SVs. Therefore, as seen in Table 4A,B, except for the black water category of flag cichlids, the number of non-zero SVs in the gut and mucus bacterial communities across other categories was similar. In the mixed water and white water datasets for flag cichlids, the mucus microbiota network exhibited a slightly higher average degree than the gut microbiota network. However, in the black water category, the mucus microbiota showed more non-zero SVs and a much higher average degree than the gut microbiota, suggesting a denser mucus microbiota network in flag cichlids. This density difference was less pronounced in the mixed water and white water categories. In contrast, for black piranhas in black water and white water, the gut microbiota network had a significantly higher average degree than the mucus microbiota network despite having a similar number of non-zero SVs. This indicates a much denser gut microbiota network in black piranhas compared to their mucus microbiota network. Therefore, our analysis using SMG suggested that gut bacterial communities were generally more interconnected than mucus bacterial communities, consistent with the findings of Sylvain et al. (2014) [13].

**Figure 3:**
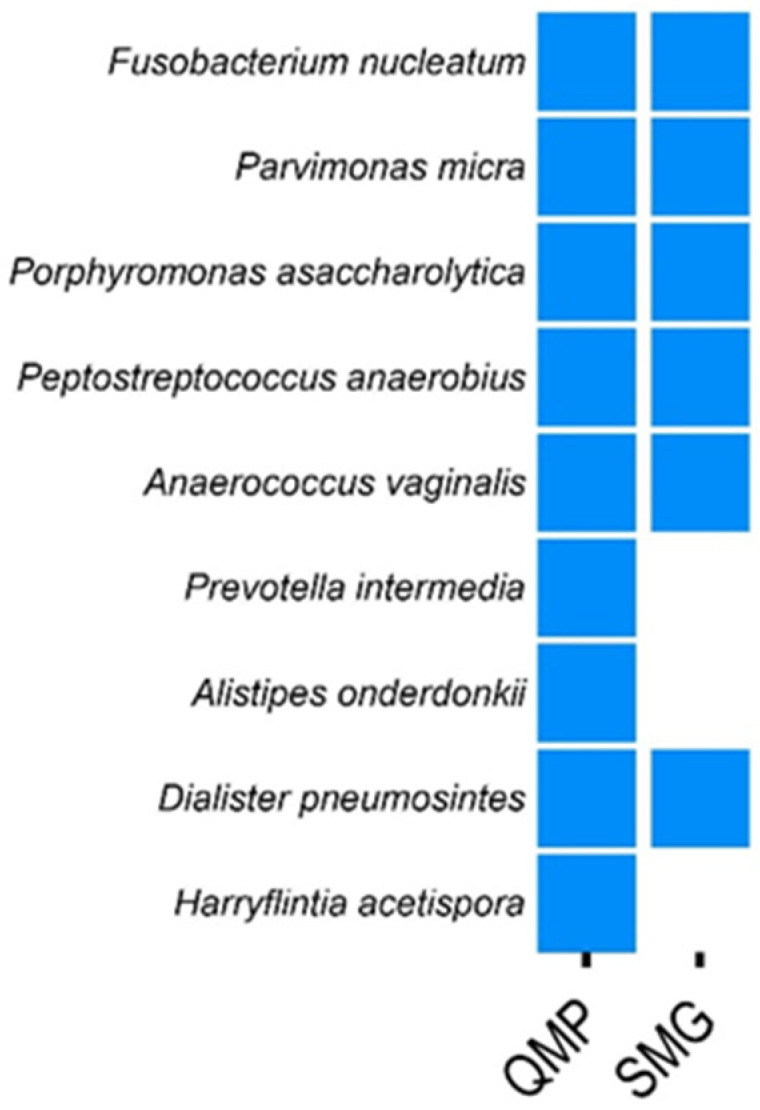
Comparison of colorectal cancer (CRC) biomarkers identified by SMG and QMP. This figure presents a comparative analysis of CRC biomarkers identified using SMG, with data sourced from Tito et al., 2019 (refer to Supplementary File S5) [14]. Tito et al. [14] originally identified nine CRC biomarkers through quantitative microbiome profiling (QMP). In our study, we confirmed six of these nine biomarkers using SMG. Additionally, SMG detected several CRC-associated species that were not identified by QMP, highlighting the potential of SMG to uncover additional biomarkers.

Using quantitative microbiome profiling (QMP), Tito et al. identified nine species associated with colorectal cancer (CRC) [14]. In our study, we applied a 99% comparison filter using SMG and confirmed six of these species across both CRC vs. ADE (patients with polyps) and CRC vs. CTL (patients without colonic lesions) comparisons (Figure 3). Additionally, SMG identified several CRC-associated species not detected by QMP but reported in other studies. These include Gordonibacter pamelaeae, Streptococcus oralis, Solobacterium moorei, and Bacteroides nordii (refer to Supplementary File S5). Therefore, our findings demonstrated that SMG was comparable to established tools like QMP in identifying CRC-associated species and may offer additional insights by detecting species overlooked by QMP.

## 4. Discussion

### 4.1. SMG Robustly Clusters the “Target of Interest”

In Table 1, SMG distinguishes the manually added “target of interest.” In contrast to SparCC, our approach exhibits sensitivity to both “positive” and “negative” relationships, showcasing a potential for effectively distinguishing closely related microbial communities. Correlation detection strategies in microbial datasets vary greatly [19], but the 100% sensitivities and specificities from SMG are stable. However, normalized covariance (correlations) almost never provides reliable biotic interactions in ecosystems [20], making further wet-lab testing necessary and valuable. Notably, even though SparCC grouped the “target of interest” rows correctly, a confirmation step may be required [2].

We highlight that the Bray–Curtis dissimilarity may inaccurately generate clusters under certain conditions, raising questions about the completeness and accuracy of conclusions drawn in previous studies if the authors relied solely on output results without a comprehensive understanding of the data characteristics. Even SparCC, designed to mitigate false correlations [17], encountered failure in 2 out of 10 simulations related to microbiological community clustering (Table 1). This underscores the necessity to enhance each tool’s stability, provide user guidance on suitable data structures, and conduct preliminary testing before inputting data into the software.

### 4.2. Advantages of Using SMG

Our SMG algorithm offers researchers a novel perspective for structurally analyzing microbiome compositional data by potentially uncovering insights that traditional algorithms based solely on taxa values might miss. This algorithm uniquely considers the rate of change (i.e., slope) between any two samples for each taxon, thereby constructing an overall structure for each taxon across all samples and computing the structural differences, known as slope-based distances, between taxa. This approach ultimately facilitates the determination of relationships between taxa.

SMG is highly versatile regarding raw data selection. It can be applied to both absolute abundance and relative abundance data, providing significant flexibility in research. Additionally, the SMG formula is straightforward and can yield results without the need for specialized software. In this current study, we used Visual Basic for Applications (VBA) built into Microsoft Excel 2023 (refer to Data Availability Statement section for example use of SMG). Since SMG does not depend on complex and costly software, it presents an affordable option for research groups with limited funding. Our provided VBA programs (see Data Availability Statement) may help validate their hypotheses and guide subsequent research.

The excess of zeros in compositional data always brings concerns. For instance, zero-inflated data may violate the assumptions of normal, Poisson or binomial distribution, making linear models cannot be used. As mentioned previously, the violation of conditions would possibly generate wrong interpretations. In addition, the overdispersion caused by zero-inflated data may also lead to false positive results [21,22]. Moreover, researchers have been exploring log transformed related methods, but potential issues, such as logarithm of zero (produce undefined values) and log-transforming compositional data can distort the relative relationships between the components [9,14]. One of the advantages of SMG is its capability to effectively handle zeros. For instance, log-ratio transformations require replacing zeroes with small pseudo-counts because zero cannot be the denominator of a fraction and, if in the numerator, is out of the domain of the logarithmic function. In contrast, SMG handles zero entries with flexibility. Particularly, SMG does not need to replace zeroes with pseudo-counts, which are non-existent values in raw data. In addition, regardless of the number of zeroes between any two microbes, the slope distance equation can compute the distance between them. Meanwhile, SMG considers the characteristics of distances between indicative points in multidimensional space, ensuring a rational analysis of subsequent matrices and graphs within positive correlation boundaries, even when a substantial number of zeroes are present.

Another overlooked phenomenon is that some experimental designs may suffer from insufficient sample sizes, which may lead to inaccurate interpretations. For instance, it is impossible to find any between-group differences if the analyzed microbiome matrix only contained two samples (the number of columns). Indeed, the previously published ANCOM tool version cannot handle <20 samples. Nonetheless, these results demonstrate that the SMG results are robust, comparable to other metrics (such as widely applied ZicoSeq and ANCOM-BC2), and able to handle zeros, alleviating concerns about violating limitations of use.

### 4.3. Recommendations on Dealing Rare Taxa Using SMG

In raw datasets, there are typically some taxa with very small absolute abundance values, usually ranging from 1 to 10 across all samples. These are commonly referred to as rare taxa. Since min–max feature scaling is applied before utilizing the slope-based distance function, the range of values for all taxa is scaled to [0, 1]. This process elevates those rare taxa to the same level as taxa with larger proportions in the dataset. While this is not a significant issue when the proportion of rare taxa is small, it can obscure significant differences when they constitute a substantial portion of the dataset.

Previous studies have highlighted the challenges posed by rare taxa in biological data analysis [23]. Our findings are consistent with these observations, emphasizing the importance of appropriate filtering techniques (Section 2.1.5). To more accurately compare the major components of bacterial compositions between two environments, we focus on the primary taxa, which account for a significant proportion of the overall environment. Therefore, we apply a comparison filter to exclude the minor components, effectively zeroing out the smallest values that cumulatively make up a very small percentage of the total data. This mitigates the impact of rare taxa, ensuring that the differences between major components are more clearly observed.

In Section 3.3 (Table 3), if we select a comparison filter between 95% and 99.5%, the sum of the number of edges in the symmetric difference graphs for both Day 24 and Day 40 data remains relatively stable. This indicates that the impact of rare taxa has been mitigated. However, without applying any comparison filters (i.e., setting the comparison filter to 100%), the results differ significantly from those obtained with other comparison filters. Notably, the influence of rare taxa in the Day 40 data is substantially greater than in the Day 24 data. In the Day 40 data, rare taxa obscure nearly all differences in the results. Therefore, applying a comparison filter with proportional zeroing is necessary when comparing biological structures in certain datasets.

In Section 3.2 (Table 2 and Table S1), SMG with a comparison filter and proportional zeroing can also be utilized in DAA. Although, as shown in Table 2, SMG can identify all the distinct OTUs in the accuracy comparisons, Table S1 indicates that SMG requires the application of a specific comparison filter to achieve the most precise results. Consequently, selecting the most suitable filter may present a challenge that needs to be addressed. However, this issue arises only when SMG is used in isolation. When combined with additional data, we can obtain relevant information to determine the optimal filter. For instance, we can estimate the number of OTU changes we expect to find by considering the average values of all samples within each OTU while examining the study data. This approach allows us to roughly delineate the range of the comparison filter. Alternatively, we can determine the optimal filter based on the structure of the results. As demonstrated in Table S1, when the DA is 3%, the optimal filter is either 97.5% or 98%. When the filter exceeds 98%, a precipitous drop in accuracy is observed, a trend that is also apparent for other DA values. Thus, when there is a significant decrease in accuracy, the point just before the decline likely represents the optimal filter. Hence, rather than relying on guesswork, there are several methods to assist in identifying the optimal filter. Additionally, this further illustrates that within comparative data, the combination of a comparison filter and proportional zeroing can effectively complement SMG.

While filtering out rare taxa when using SMG may improve accuracy in comparing datasets, it is undeniable that some rare taxa play significant roles in data analysis [24]. Therefore, in our analysis of individual datasets (Section 3.1), all data, including rare taxa, were retained. The inclusion of rare taxa information allowed us to achieve 100% accuracy in analyzing simulation data type 1. Consequently, we recommend that when using SMG to analyze a single dataset, all information, including rare taxa, should be preserved. Conversely, when using SMG for comparative analysis across multiple datasets, the application of a comparison filter with proportional zeroing (Section 2.1.5) can significantly enhance SMG accuracy.

### 4.4. Importance of Using SMG in Microbiome Data Analysis

The SMG algorithm not only offers a novel approach to microbiome data analysis but also addresses critical challenges in the field, such as the handling of zero-inflated data and the accurate clustering of microbial communities. These advancements have significant implications for various domains that rely on microbiome research, from human health to environmental science. By providing a more reliable tool for analyzing complex microbial ecosystems, SMG could enhance our understanding of the role of microbiomes in health and disease, support the development of targeted therapeutic interventions, and inform sustainable practices in agriculture. Furthermore, SMG’s straightforward implementation and cost-effectiveness make it accessible for broader use, potentially setting a new standard in microbiome analysis. As the field continues to evolve, SMG’s capacity to integrate with other omics data and track microbiome dynamics over time offers avenues for future research, positioning it as an asset in the growing toolkit of microbiome scientists.

### 4.5. Limitations of Use

Although SMG has advantages, it also exhibits limitations. Currently, SMG algorithm-related formulas are calculated using the Microsoft Excel VBA, and when the sample size of the entire microbiome is large, the computational burden increases significantly for Microsoft Excel. Specifically, if we have a microbiome containing 2000 taxa with 100 samples, computing the entire distance matrix requires 2000^2^ × 100^2^, which is 40 billion calculations. While Excel can technically perform billions of calculations, it requires sufficient hardware, CPU, available RAM, calculation complexity, and spreadsheet optimization. Therefore, when using Excel in conjunction with SMG for data analysis, we currently recommend limiting the number of taxa to 500 and the number of samples to 50, keeping the computation time within a relatively manageable range. Further programming development is underway, e.g., using programming languages such as Python or Perl to increase computing capacity. For example, libraries like NumPy, SciPy, and pandas provide optimized functions for working with large datasets and performing matrix operations.

## 5. Conclusions

In this study, we introduced the Slope-Matrix-Graph (SMG) algorithm for analyzing microbiome compositional data, with a focus on handling zero-inflated data and clustering microbial communities using slope-based distances. Our evaluation using simulated datasets showed that SMG outperforms traditional methods, like Bray–Curtis dissimilarity and SparCC, by accurately clustering microbes into distinct positive and negative correlation groups, achieving 100% sensitivity and specificity in identifying “targets of interest” within microbiome data, which highlights its robustness and reliability. SMG’s approach to filtering rare taxa enhances the accuracy of differential abundance analysis (DAA), allowing for clearer identification of major taxonomic differences, with potential to advance our understanding of microbial communities. 

Furthermore, our analysis revealed that SMG’s approach to filtering rare taxa using a comparison filter with proportional zeroing can significantly improve the accuracy of DAA. This method allows for clearer observation of major taxonomic differences, especially in datasets where rare taxa might otherwise obscure meaningful patterns.

Overall, SMG offers a powerful and versatile framework for microbiome analysis, with significant potential to advance our understanding of microbial community structures. By providing a more accurate and accessible method for analyzing microbiome data, SMG contributes to the broader goals of improving microbiome research accuracy and facilitating new discoveries in microbial ecology. However, the computational demands of SMG, particularly when dealing with large datasets, present a current limitation. Future work will focus on optimizing SMG for large-scale data analysis and exploring its application in a wider range of microbiome studies.

## Figures and Tables

**Table 1:** Compared method performance summary. ^#^ with non-designated correlation data points clustered in; * two 50 × 10 matrix failed in generating any clusters by SparCC.

Method	Positive Correlation	Negative Correlation	Reproducibility
Bray–Curtis Dissimilarity	Network node chain contains only positive correlations from coefficients of 0.1 to 1	None	10/10
SparCC	Correctly grouped ^#^	Correctly grouped ^#^	8/10 *
SMG	Correctly grouped	Correctly grouped	10/10

**Table 2:** Accuracy comparisons of differential abundance analysis (DAA) tools. Note: ANCOM-BC2 cannot handle ≤ 20 microbiome “rows”; ^#^ parameter calculated based on ≥5% graph edge changes; ^$^ 0.955 filter threshold used instead of 0.975 filter applied on differential abundance from 3% to 10%.

	Accuracy (TP + TN)/(TP + TN + FP + FN)		
DA%	ZicoSeq	ANCOM-BC2	SMG-Graph ^#^
3%	0.88	1.00	1.00
5%	0.97	1.00	1.00
7%	1.00	1.00	1.00
10%	0.99	0.99	1.00
20%	0.98	0.94	1.00 ^$^

**Table 3:** Symmetric difference results of published data revisit for chicken gut microbiota in response to antibiotic growth promoters (AGPs) study [12].

A							
Day 24 Filter	C vs. W	C+ vs. W+	Change Diet	C vs. C+	W vs. W+	Change AGP	Difference
95%	12,292	9525	21,817	10,369	6981	17,350	4467
95.5%	12,680	10,148	22,828	10,581	7307	17,888	4940
96%	12,924	10,743	23,667	10,918	7234	18,152	5515
96.5%	13,131	10,939	24,070	10,815	8254	19,069	5001
97%	13,751	11,299	25,050	10,530	7817	18,347	6703
97.5%	14,169	11,448	25,617	10,673	7897	18,570	7047
98%	14,510	12,029	26,539	10,717	8660	19,377	7162
98.5%	14,582	12,364	26,946	10,688	8985	19,673	7273
99%	14,135	13,388	27,523	10,406	9209	19,615	7908
99.5%	14,708	14,074	28,782	10,658	10,261	20,919	7863
100%	11,499	8229	19,728	9623	6821	16,444	3284
B							
Day 40 filter	C vs. W	C+ vs. W+	Change Diet	C vs. C+	W vs. W+	Change AGP	Difference
95%	16,345	10,764	27,109	11,679	8570	20,249	6860
95.5%	16,394	10,891	27,285	11,832	9327	21,159	6126
96%	16,366	10,784	27,150	11,480	9068	20,548	6602
96.5%	15,618	10,509	26,127	11,222	9131	20,353	5774
97%	15,543	10,305	25,848	11,095	9215	20,310	5538
97.5%	14,819	10,089	24,908	10,435	9619	20,054	4854
98%	14,142	9255	23,397	9848	8739	18,587	4810
98.5%	13,060	9255	22,315	9043	8806	17,849	4466
99%	11,777	8770	20,547	7733	8110	15,843	4704
99.5%	10,091	7570	17,661	6390	7377	13,767	3894
100%	4057	4238	8295	3142	4941	8083	212

**Table 4:** Average degree of networks for gut and skin mucus bacterial communities in flag cichlid and black piranha with different water environments (revisit of published data from [13]).

A						
Gut	Flag Cichlid			Black Piranha		Total
	Black Water	Mixed Water	White Water	Black Water	White Water	
Non-zero ASV	698	718	2695	1844	3138	7383
Average Degree	31.14	44.21	184.62	295.63	233.73	247.82
B						
Mucus	Flag Cichlid			Black Piranha		Total
	Black Water	Mixed Water	White Water	Black Water	White Water	
Non-zero ASV	1868	804	2655	1765	2836	7998
Average Degree	116.03	69.59	225.64	166.64	127.46	190.97

## Data Availability

The raw data supporting the conclusions of this article will be made available by the authors on request.

## Supplementary Materials

The following supporting information can be downloaded at: https://www.mdpi.com/article/10.3390/microorganisms12091866/s1, File S1: A systematic investigation into the composition and functionality of chicken gut microbiota; File S2: Simulation data type 1 and the clustering results; File S3: Simulation data type 2 and the DAA results; File S4: Amazon fish raw SV and metadata; File S5: List of fecal microbiota of 589 patients across three colorectal cancer (CRC); Figure S1: Overview of relative abundance data of simulation type 1 (an example of 100 × 20 matrix); Table S1: Zeroing filter impact on SMG’s performances. DA: differential abundance; note: 3% to 10% DA percentages show similar performance trends (only showing 3% as an example), but 20% DA differs with others.

## Abbreviations

NGS	next-generation sequencing
SparCC	sparse correlations for compositional data
ANCOM-BC2	analysis of composition of microbiomes with bias correction 2
SMG	slope-matrix-graph
DAA	differential abundance analysis
OTUs	operational taxonomic units
SVs	sequence variants
FDR	false discovery rate
GT	graph theory
