# Supplementary material for: A Novel Slope-Matrix-Graph Algorithm to Analyze Compositional Microbiome Data
Source: Microorganisms. 2024 Sep 9;12(9):1866. doi: 10.3390/microorganisms12091866 (PMC11434172; doi:10.3390/microorganisms12091866)
Supplement: Supplementary file 1 [file microorganisms-12-01866-s001.zip › microorganisms-3174079-supplementary.pdf]

microorganisms

MDPI

A Novel Slope-Matrix-Graph Algorithm to Analyze Compositional Microbiome Data

Meng Zhang [1], Xiang Li [2,*], Adelumola Oladeinde [2], Michael Rothrock Jr. [2], Anthony Pokoo-Aikins [3] and Gregory Zock [2]

[1] University of North Georgia, Department of Mathematics, 82 College Cir, Dahlonega, GA 30597, USA
[2] U.S. National Poultry Research Center, Egg & Poultry Production Safety Research Unit, Agricultural Research Service, U.S. Department of Agriculture, 950 College Station Road, Athens, Georgia 30605, USA
[3] U.S. National Poultry Research Center, Toxicology & Mycotoxin Research Unit, Agricultural Research Service, U.S. Department of Agriculture, 950 College Station Road, Athens, Georgia 30605, USA
* Correspondence: xiang.li@usda.gov

Supplementary Figure and Table

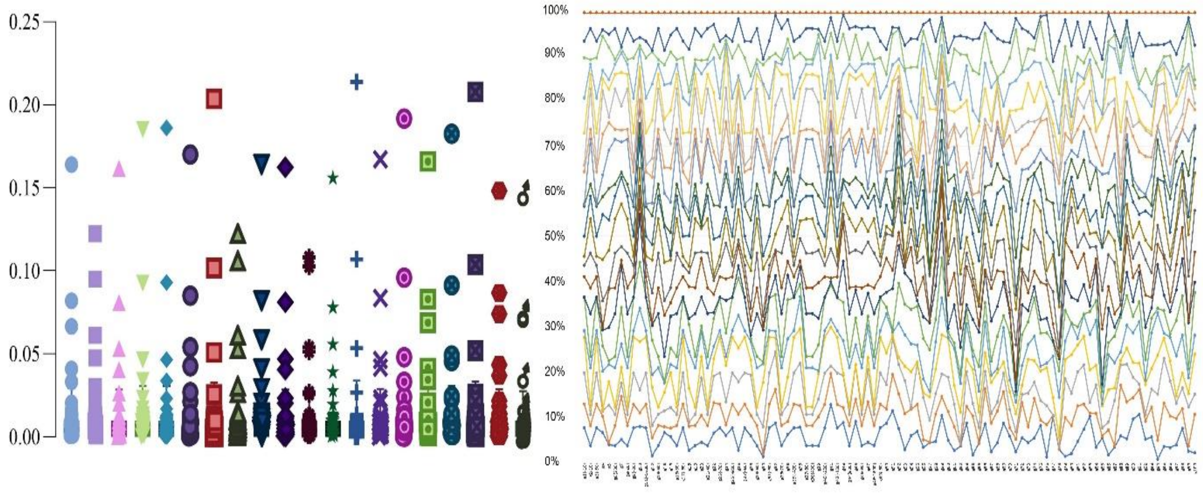

Figure S1. Overview of relative abundance data of simulation type 1 (an example of 100 × 20 matrix).

Table S1. Zeroing filter impact on SMG's performances. DA: differential abundance; note: 3% to 10% DA percentages show similar performance trends (only showing 3% as an example), but 20% DA differs with others.

DA%.	Filter threshold	Accuracy
3.00%	0.950	95.00%
	0.955	96.20%
	0.960	97.10%
	0.965	97.10%
	0.970	98.00%
	**0.975**	**100.00%**
	**0.980**	**100.00%**
	0.985	0.00%
	0.990	0.00%
	0.995	0.00%
	1.000	0.00%

DA%	Filter threshold	Accuracy
20.00%	0.950	99.00%

	**<u>0.955</u>**	**<u>100.00%</u>**
	0.960	<100.00%
	0.965	<95.00%
	0.970	<60.00%
	0.975	<30.00%
	0.980	<20.00%
	0.985	0.00%
	0.990	0.00%
	0.995	0.00%
	1.000	0.00%
